# Cubic-Phase Metasurface for Three-Dimensional Optical Manipulation

**DOI:** 10.3390/nano11071730

**Published:** 2021-06-30

**Authors:** Hsin Yu Kuo, Sunil Vyas, Cheng Hung Chu, Mu Ku Chen, Xu Shi, Hiroaki Misawa, Yu-Jung Lu, Yuan Luo, Din Ping Tsai

**Affiliations:** 1 Department of Physics, National Taiwan University, Taipei 10617, Taiwan; d06245003@ntu.edu.tw (H.Y.K.); yujunglu@gate.sinica.edu.tw (Y.-J.L.); 2 Research Center for Applied Sciences, Academia Sinica, Taipei 11529, Taiwan; cheng-hung.chu@riken.jp; 3 Institute of Medical Device and Imaging, National Taiwan University, Taipei 10051, Taiwan; sunilvyas@ntu.edu.tw; 4 Department of Electronic and Information Engineering, The Hong Kong Polytechnic University, Hong Kong 999077, China; mu-ku.chen@polyu.edu.hk; 5 Research Institute for Electronic Science, Hokkaido University, Sapporo 001-0021, Japan; shixu@es.hokudai.ac.jp (X.S.); misawa@es.hokudai.ac.jp (H.M.); 6 YongLin Institute of Health, National Taiwan University, Taipei 10672, Taiwan; 7 Department of Biomedical Engineering, National Taiwan University, Taipei 10051, Taiwan

**Keywords:** dielectric metasurface, vertically accelerated 2D Airy beam, 3D optical manipulation

## Abstract

The optical tweezer is one of the important techniques for contactless manipulation in biological research to control the motion of tiny objects. For three-dimensional (3D) optical manipulation, shaped light beams have been widely used. Typically, spatial light modulators are used for shaping light fields. However, they suffer from bulky size, narrow operational bandwidth, and limitations of incident polarization states. Here, a cubic-phase dielectric metasurface, composed of GaN circular nanopillars, is designed and fabricated to generate a polarization-independent vertically accelerated two-dimensional (2D) Airy beam in the visible region. The distinctive propagation characteristics of a vertically accelerated 2D Airy beam, including non-diffraction, self-acceleration, and self-healing, are experimentally demonstrated. An optical manipulation system equipped with a cubic-phase metasurface is designed to perform 3D manipulation of microscale particles. Due to the high-intensity gradients and the reciprocal propagation trajectory of Airy beams, particles can be laterally shifted and guided along the axial direction. In addition, the performance of optical trapping is quantitatively evaluated by experimentally measured trapping stiffness. Our metasurface has great potential to shape light for compact systems in the field of physics and biological applications.

## 1. Introduction

Optical tweezer [1,2,3] has become an indispensable tool to operate the motion of micro- or nanoscale objects for biomedical research [4,5,6,7]. Because of a non-contact approach, the manipulation technique can hold, drag and even twist objects without mechanical damage and has been widely used in diverse applications ranging from the biomedical analysis [4,8,9] to micro-robot engineering [10]. In the conventional configuration, an optical tweezer is driven by a focused Gaussian beam to generate optical gradient force to confine particles to only a few micrometers range due to short Rayleigh lengths [11]. To expand the freedom of stable operation from one dimension to three dimensions, optical manipulation systems based on two opposite laser beams [12], two crossed laser beams [13], and non-diffraction beams [14,15,16,17] have been demonstrated and applied in physical [10,18,19,20,21], chemical [22,23], and biological [6,7,8,9] sciences. Compared with Gaussian beams, non-diffraction and self-healing beams, such as the Bessel beam, Airy beam, and abrupt autofocusing beam, are able to maintain propagation properties up to long propagation distances [24]. The Airy beam has spatially asymmetric intensity distribution, parabolic propagation trajectory, and self-acceleration behavior, which was theoretically found [25] by Berry and Balazs in 1979 and experimentally observed [26] by Siviloglou et al. in 2007. In recent years, Airy beams have attracted large interest in various applications, including the generation of curved plasmonic channels [27], micromachining of curved profiles [28], and 3D super-resolution imaging [29,30]. In optical manipulation techniques, particularly, Airy beams exhibit unique optical properties and have been employed for optical snow blowers [14] and optical conveyors [17,31], including guiding, pushing, and trapping. Traditionally, an Airy beam is generated by a dynamic diffractive element, such as a spatial light modulator, deformable mirror, or digital micro-mirror device (DMD), which suffers from bulky size, redundant diffraction orders, and low transmission efficiency.

Metasurface [32], which consists of metal or dielectric subwavelength structures to modulate the phase, polarization, or amplitude of electromagnetic waves at subwavelength levels, has attracted attention in nanophotonic devices [33,34,35,36,37]. With flexible, compact, and flat geometry, metasurfaces can provide a tailored design for miniature systems for various applications [38,39,40,41,42]. Recently, generating Airy beams based on metasurface has been introduced [43]; however, a finite number of designed parameters for composed unit cells are inevitably limited by a constant period and fabrication feasibility such that phase modulation is insufficient to cover an entire 2π regions [44]. Some previous works sacrifice operation bandwidth, size or transmission efficiency to overcome the entire 2π phase issue [45,46,47]. Alternatively, geometric phase methods [48] (also known as Pancharatnam–Berry phase methods) may be applied to provide additional phase compensation to arrange different rotation angles for each unit cell. Although it can relax the problem of inadequate phase modulation, the geometric phase-based metasurfaces only operate under certain polarization states of incident light [47,49,50,51,52,53].

Here, we present a polarization-independent cubic-phase dielectric metasurface for the generation of a vertically accelerated 2D Airy beam with high efficiency in the visible region. The fabricated metasurface is composed of GaN circular nanopillars that provides low loss, high robustness, and high refractive index over the entire visible spectrum, which would excite multipole waveguide-like cavity resonant modes in nanopillars [36] and implement the entire 2π phase coverage. The propagation characteristics of the Airy beam are further analyzed with numerical calculations and finite-difference time-domain (FDTD; Lumerical Solutions, Inc., Vancouver, BC, Canada) simulation. The experimental propagation trajectory of a generated Airy beam demonstrates a reciprocal curve in free space, and matches theoretical design and simulation prediction. Moreover, we integrate the metasurface into an optical manipulation system to guide and trap microparticles along the propagation path of the vertically accelerated Airy beam. Due to the high-intensity gradient created by the vertically accelerated Airy beam, attracted particles are not only laterally shifted, but also trapped in the axial direction. The trapping stiffness of the system has been evaluated to quantify the performance of optical manipulation. The dynamic motion of attracted microparticles has been real-time observed in 3D. In our knowledge, this is the first report on the realization of 3D optical manipulation based on a cubic-phase metasurface.

## 2. Methods

**Figure 1:**
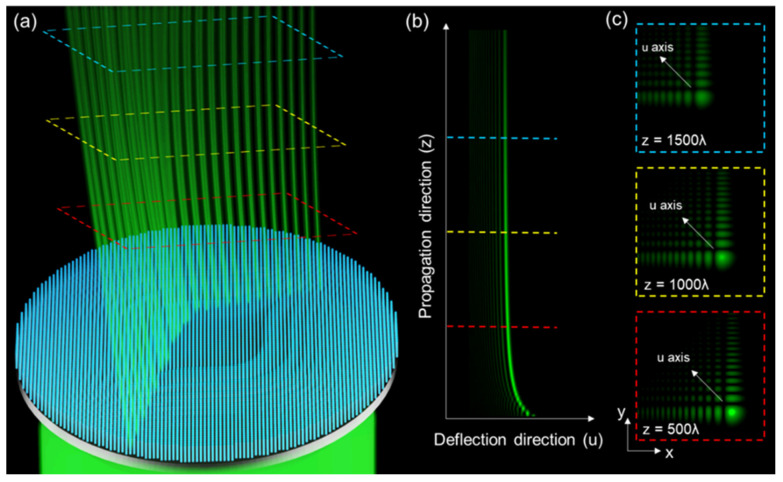
Propagation characteristics of a vertically accelerated 2D Airy beam. (**a**) Schematic diagram of generating a vertically accelerated 2D Airy beam by an all-dielectric cubic-phase metasurface. (**b**) Numerically calculated beam trajectory of a vertically accelerated 2D Airy beam along the *u-z* plane and the cross-section of intensity distribution at different propagation planes. (**c**) The deflection of the main lobe of a vertically accelerated 2D Airy beam is along the diagonal direction of the *x-y* plane, denoted as the *u-*direction.

A transmission-type dielectric metasurface is designed to generate a vertically accelerated 2D Airy beam with the inherent properties of non-diffraction, self-bending, and self-healing. A schematic diagram of the metasurface is shown in Figure 1a. In contrast to a paraxial wave equation of Airy beams [25,51], the phase distribution of the metasurface is calculated based on the principle of geometrical optics [47,49]. One-dimensional trajectory curve, reduced from 2D trajectory plane of accelerating beam with the metasurface positioned on *z* = 0 plane, is used for simplicity. The reciprocal propagation trajectory curve of the accelerating beam is *z* = f (*x*), and (*x*_0_, *z*_0_) is an arbitrary position along the trajectory curve. According to the principle of geometric optics [47], the tangential equation of the position can be indicated as *z* = f′ (*x*) (*x* − *x*_0_) + *z*_0_ with the intersection point of tangent and metasurface is *x* = *x*_0_ − *z*_0_/f′(*x*_0_). The phase difference between the intersection point of (*x*_0_, *z*_0_) and the minimized optical path from the metasurface is dϕ(*x*) = (*x* − *x*_0_)∙sin*θ∙k*_0_, where *θ* is the angle between the tangent and optical axis, *k*_0_ = 2*π*/*λ* is the magnitude of the wave vector of light and *λ* is the vacuum wavelength. In the paraxial approximation, sin*θ* can be written as d*x*/*z*_0_ so that the phase gradient along the plane of the metasurface is

dϕ(*x*)/d*x* = −*k*_0_/f′(*x*_0_). (1)

assuming that the formula of reciprocal propagation trajectory for parameter *x* and *y* is independent. The formula of 2D accelerating beam curve is set as $\left\{\begin{array}{l} z=a/x\\ z=a/y \end{array}\right.$, where *z* can have two equations with a as trajectory constant. According to Equation (1), the phase distribution of the metasurface can be described as:

ϕ(*x*, *y*) = *k*_0_ (*x*^3^ + *y*^3^)/12*a*. (2)

The expression of the cubic-phase profile from the principle of geometrical optics is identical with the phase distribution of the vertically accelerated 2D Airy beam from paraxial Helmholtz equation [26,54]. In this work, the trajectory constant was set a = 0.033 mm^2^ and the operation wavelength λ = 532 nm. To validate the propagation characteristics of the Airy beam generated by cubic phase, we numerically calculated the intensity distribution along the optical axis at the different propagation distances, as shown in Figure 1b,c, respectively. It is noteworthy that the deflection direction of the main lobe of a vertically accelerated 2D Airy beam is along the diagonal direction of the *x* and *y-*axis, which are also denoted as the *u-*direction.

## 3. Results

### 3.1. Simulated Results and Sample Fabrication

**Figure 2:**
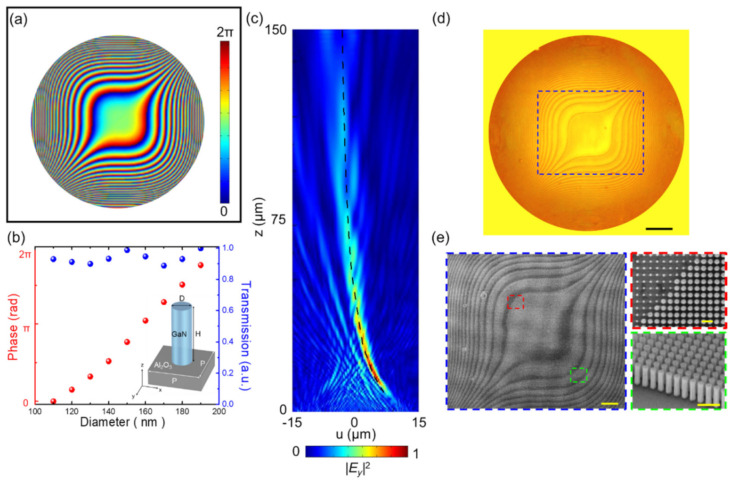
Design, simulation, and fabrication of a vertically accelerated 2D Airy beam generated by a cubic-phase metasurface. (**a**) Phase distribution of the cubic-phase metasurface. (**b**) The cubic-phase metasurface is composed of GaN circular nanopillars with a height of 800 nm and a pitch (P) of 250 nm on an Al_2_O_3_ substrate. The corresponding phase delay level and transmission of GaN circular nanopillars are 110 nm to 190 nm diameter (D). (**c**) FDTD simulated the intensity distribution profile of the GaN metasurface with a cubic phase along the optical axis at the operating wavelength of 532 nm under y-polarized incident light. The black dash line shows the theoretical propagation trajectory of the cubic-phase metasurface. (**d**) Photographic image of the fabricated cubic-phase metasurface. Scale bar: 50 µm. The blue dashed square denotes the region of the SEM image shown in (**e**). (**e**) SEM image of the center position of the fabricated metasurface. Scale bar: 20 µm. Top (highlighted in a red square) and tiled views (highlighted in a green square) of a zoomed-in SEM image of GaN circular nanopillars. Scale bar: 0.5 µm.

According to Equation (2), the phase distribution of the cubic-phase mask for the metasurface is shown in Figure 2a. The metasurface is composed of GaN circular nanopillars with a height of 800 nm on an Al_2_O_3_ substrate. The real and imaginary permittivity of GaN can be found in Figure S1. Commercial software (CST Studio Suite, Simulia, Johnston, IA, USA) is used to simulate the phase delay level and transmission of GaN circular nanopillars at different diameters, ranging from 110 nm to 190 nm, with a pitch of 250 nm. The corresponding table of the phase delay level and transmission in unit cells with different diameters can be found in Tables S1–S3. The intensity distribution (|*Ey*|^2^) in the diffracted field from GaN dielectric metasurface with cubic phase at 532 nm is demonstrated by the FDTD method. More detailed information about simulated parameters and boundary conditions can be found in the Supplementary Materials in Sections S1 and S2. Simulated intensity distribution of the vertically accelerated 2D Airy beam generated by a cubic-phase metasurface in free space is shown in Figure 2c. The main lobe of the beam follows the theoretically predicted propagation trajectory (*z* = $\sqrt{2}$*a*/*u*). Since the unit cell of metasurface constitutes by circular nanopillars, regardless of the polarization direction of the incident light, the cubic-phase metasurface produces a vertically accelerated 2D Airy beam. The comparison of the propagation profiles of a vertically accelerated 2D Airy beam under different incident polarization directions can be found in Figure S2.

To fabricate our cubic-phase metasurface, we used a metalorganic chemical vapor deposition (MOCVD) and a plasma-enhanced chemical vapor deposition (PECVD) process to deposit 800 nm of GaN thin film on a double-polished Al_2_O_3_ substrate and 400 nm of silicon dioxide (SiO_2_) as a hard mask layer, respectively. Using a spin coater, the diluted positive photoresist (ZEP-520A:ZEPA = 1:1) layer of ~150 nm was grown onto the hard mask layer at room temperature. Afterward, an electron beam lithography system was used to draw the designed cubic-phase pattern on the coated substrate. The pattern was developed by the developer solution (ZED-N50) and formed onto a SiO_2_ hard mask layer by reactive ion etching (RIE), in which an etching hard mask of ~35 nm Cr thin film was used. The GaN circular nanopillars were framed by ICP-RIE with BCl_3_/Cl_2_ mixtures. After cleaning a residual SiO_2_ hard mask, the GaN metasurface with the cubic-phase distribution was successfully fabricated [55]. Figure 2d,e, respectively, show the photographic image and the scanning electron microscope (SEM) images of the fabricated cubic-phase metasurface with a 0.8 mm aperture diameter.

### 3.2. Experimental Verification of Propagation Characteristics of Generated Airy Beam

**Figure 3:**
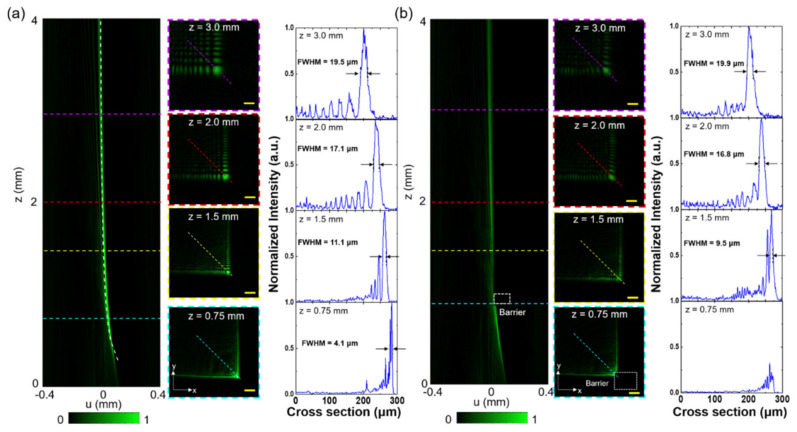
Experimental verification of propagation properties for a vertically accelerated 2D Airy beam generated by a cubic-phase metasurface. Measured intensity distribution for propagation trajectory of the vertically accelerated 2D Airy beam in free space (**a**) without or (**b**) with a barrier at *z* = 0.5 mm at 532 nm. The theoretical propagation trajectory is indicated as the white dashed line. The cross-sectional normalized intensity distributions of the propagation profile of a vertically accelerated 2D Airy beam. The corresponding cross-sectional image of intensity distribution at propagation positions of *z* = 0.75, 1.5, 2.0 and 3.0 mm, respectively. Scale bar: 25 µm. The intensity profiles of the main lobe of the beam along u-direction (dash line) are at *z* = 1.5, 2.0, and 3.0 mm.

The experimentally measured intensity distribution of the cubic-phase metasurface along the propagation direction at the designed wavelength λ = 532 nm is shown in Figure 3a. The metasurface shapes the incident Gaussian beam into a vertically accelerated 2D Airy beam and the main lobe of generated beam follows the theoretically predicted propagation trajectory indicated by a white dashed line. More details about measurements can be found in Figure S3. The generated vertically accelerated 2D Airy beam offers a large working distance and shows good agreement with the theoretical prediction, despite the insufficient deflection of the beam propagation trajectory near the surface. The deviation between experimental and theoretical results for propagation appears due to the defects of the sample, which causes phase distortion in the resultant beam. Along the propagation direction, it is obvious that the diffraction patterns transform from cubic-phase mask at *z* = 0.0 mm to form inherent *L*-shape intensity pattern at *z* = 0.75 mm, and finally to produce a 2D Airy field distribution at *z* = 1.5 mm, with the main lobe and multiple side lobes in both *x* and *y* directions. The position of the main lobe is shifted along the *u-*direction, and its size is increased during propagation. The dynamic propagation behavior of a vertically accelerated 2D Airy beam is quantitatively analyzed in the normalized intensity profiles of cross-sectional field distribution (along the yellow dashed line), as shown in Figure 3a. The propagation distance of the beam is around 4 mm which can be further extended with different metasurface design parameters. The average transmission efficiency of the metasurface, which is defined as the ratio of the power of the generated beam to the power of input light, is ~40% at the operating wavelength of 532 nm. Because of GaN circular nanopillars, the metasurface is not limited in its operation by the polarization states of incident light. To validate the polarization-independent characteristics of the metasurface, a Gaussian beam with *x-* and *y-*polarization is used to generate a vertically accelerated 2D Airy beam by the metasurface, respectively. The experimental results and detailed discussions of the polarization-independent characteristics can be found in Figure S3. Because of the broadband resonance spectrum in GaN nanopillars, the fabricated metasurface has its intrinsic dispersion property which allows the metasurface to operate in the entire visible region. According to Equation (2), if the parameters of the phase distribution of the designed metasurface are constant (2*π*/12*aλ* = *c*), the operating wavelength (*λ*) is inversely proportional to the trajectory constant a. Propagation trajectories of the generated beam were measured in free space at λ = 633 nm and 491 nm, respectively. (More detailed information can be found in Figure S4). Besides the non-diffraction and self-acceleration trajectory, the self-healing behavior is one of the distinctive properties of the vertically accelerated Airy beam. In Figure 3b, to demonstrate the self-healing property, an opaque barrier, using a metal block coating black paint with a diameter of 50 μm, is positioned in the propagation path of the generated beam at *z* = 0.75 mm. The main lobe of the beam gradually reverts into the initial pattern during propagation. The self-healing property is quantitatively assessed by the cross-sectional normalized intensity profiles of field distribution at different propagation planes (*z* = 1.5 mm, 2.0 mm, and 3.0 mm) along *u-*direction. Regardless of blocking with an opaque barrier, the propagation trajectories still follow the designed propagation path. Under experimental conditions with and without a barrier in the propagation path of the beam, the difference of both deflections offset and the measured full width at half maximum (FWHM) values of the normalized intensity distribution of the main lobe are very minimal.

### 3.3. 3D Optical Manipulation

**Figure 4:**
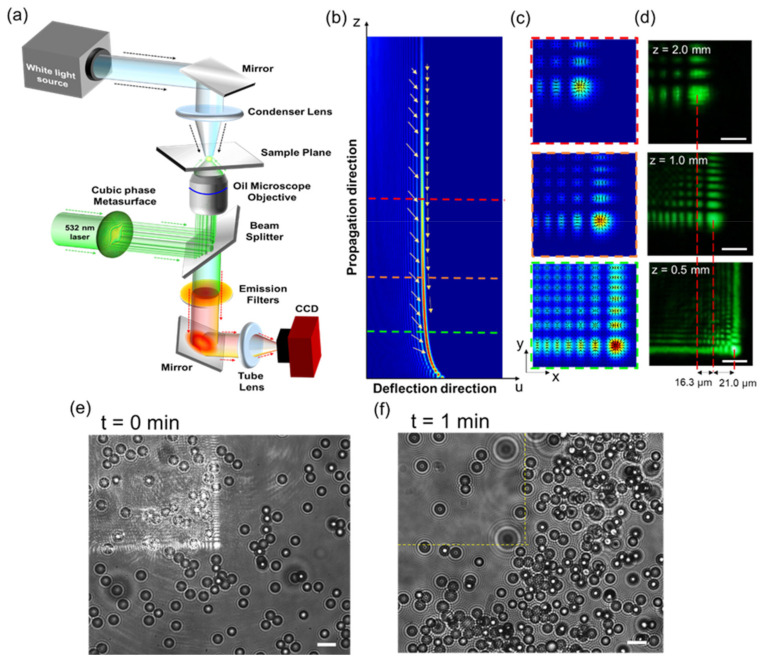
Experimental demonstration of 3D optical manipulation. (**a**) Experimental configuration of optical manipulation by the vertically accelerated 2D Airy beam, generated by a cubic-phase metasurface. Numerically calculated intensity gradient distribution of a vertically accelerated 2D Airy beam. (**b**) Along the longitudinal (*u-z*) plane, the yellow arrows represent the predicted motions of guided particles. (**c**) Along the transverse (*x-y*) planes at the different propagation depths, the black arrows indicate the magnitude and direction of the intensity gradient. (**d**) The experimental intensity results of the shifted main lobe of the beams are at *z* = 0.5 mm, *z* = 1.0 mm, and *z* = 2.0 mm, respectively. Scale bar: 25 μm. The distribution of 3 μm microspheres suspended in water solution, (**e**) before and (**f**) after illuminated with the focused beam. Scale bar: 6 μm. The yellow dash lines highlight the illuminated location of the beam.

To demonstrate the 3D optical manipulation of particles using the vertically accelerated 2D Airy beam generated from our cubic-phase metasurface in Figure 4a, a Gaussian beam with the wavelength of 532 nm (Cobolt Samba^TM^, HÜBNER Photonics, Kassel, Germany) is used. The beam is focused by an oil-immersion objective lens (Olympus UPLFLN 100X, NA = 1.4, Olympus Corporation, Tokyo, Japan) to generate sufficient power density in the sample plane, as shown in Figure 4a. For image acquisition, a white light source (EQ-99XFC LDLS^TM^, Energetiq, Wilmington, NC, USA) is focused using a condenser lens to illuminate the sample to maintain collimated light after passing through the oil-immersion objective lens. A spectral emission filter (MF630-69, Thorlabs, Newton, MA, USA) is used to separate the illumination laser light from white light and is imaged by a charge-coupled device (CCD, Prosilica GE1650, Allied Vision, Stadtroda, Germany).

The magnitude of the intensity gradient force onto trapped particles is proportional to the gradient of the intensity distribution of the focused beam and can be written as $\vec{F}\propto \vec{\nabla }{\left|E\right|}^{2}$, where *E* is the electric field of the beam [2,56]. The intensity gradient of a vertically accelerated 2D Airy beam along the transverse (i.e., *x-y* plane) and longitudinal planes (i.e., *u-z* plane) at different propagation planes is numerically calculated and shown respectively in Figure 4b–c, respectively, where the highest intensity gradient is obtained along the main lobe. In Figure 4c, the black arrows show the high-intensity gradient at the center of the main lobe. Due to the self-acceleration behavior of the vertically accelerated Airy beam, the main lobe follows the reciprocal path in the axial direction [57]. Figure 4c shows intensity distribution in the different axial planes and the center of the main lobe is shifted in the lateral direction (*x-* and *y-*direction) at different depths. Figure 4d shows the experimentally measured intensity distribution at the different propagation planes. The shifted distance of the main lobe is around 40 µm in the lateral direction with respect to the initial plane. The axial separation between the initial and final plane is around 1.5 mm. Figure 4e shows a bright-field image of a random distribution of polymer microspheres (Polybead^®^ Microspheres 3.0 µm in diameter, Polysciences, Warrington, FL, USA), suspended in a 2 mm thick slab of water. Removing the spectral filter, the intensity profile of the reflected beam is displayed on the sample plane with low input power. Once the input power reaches the threshold power for optical guiding (~170 mW), microspheres are attracted towards the main lobe of the beam in a transverse plane, and guided along the propagation trajectory of the beam in the longitudinal direction into the deep layers. As a result, the motion of attracted microspheres follows the intensity gradient distribution of the vertically accelerated 2D Airy beam, and the experimental results agree well with numerical simulations of the intensity gradients of the beam. The real-time videos of the dynamic movement of microspheres can be further found in the Supplementary Materials (Video S1). Due to the asymmetry of the intensity distribution of the vertically accelerated Airy beam, after illuminating the sample, the distribution of microspheres changes in different regions and the number of beads in the exposed areas decreases with time, as shown in Figure 4f.

**Figure 5:**
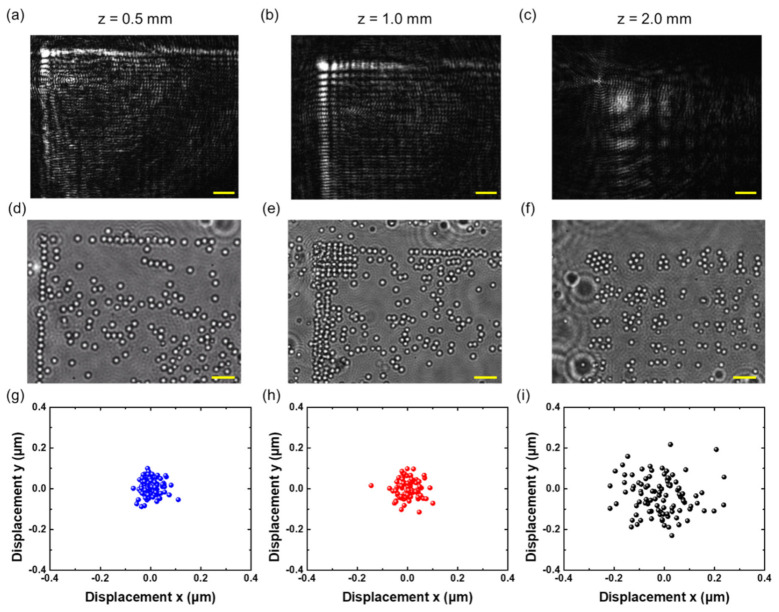
Optical trapping dynamics of microspheres based on a vertically accelerated 2D Airy beam generated by the cubic-phase metasurface. The intensity distribution of the vertically accelerated Airy beam in water along the propagation direction at (**a**) *z* = 0.5 mm, (**b**) 1.0 mm and (**c**) 2.0 mm, respectively. (**d**–**f**) After illuminating the focused beam, the corresponding distribution of microspheres appears at different depths. Scale bar: 6 μm. (**g**–**i**) Displacement of the captured microspheres around the main lobe of the Airy beam in *x-* and *y-*direction at different propagation depths with a measurement duration of 15 s.

The guiding capability of microspheres of different sizes is further demonstrated using the vertically accelerated 2D Airy beam. In this work, 1-μm microspheres (Fluoresbrite^®^ YG Microspheres 1.0 µm in diameter, Polysciences, Warrington, FL, USA) are suspended in a 20 μm thick slab of water. Figure 5a–c shows the intensity distribution of the vertically accelerated 2D Airy beam at three different depths inside the water. Similar to Figure 4b, lateral shifts of the intensity distribution at three different depths are clearly observed. Using the focused beam with an incident power of ~170 mW, the microspheres are attracted by intensity gradients and aligned in the high-intensity region. In addition, since the particle size is small, 1-μm microspheres are clustered within the inherent *L*-shaped patterns of a vertically accelerated 2D Airy beam at the bottom of the sample slab. The images of trapped microspheres are acquired by shifting the metasurface at different depths of *z* = 0.5 mm, *z* = 1.0 mm, and *z* = 2.0 mm, as respectively shown in Figure 5d–f. The experimental results of the motion from trapped microspheres along the measured propagation trajectory of a vertically accelerated 2D Airy beam imply that a long optical channel for particle manipulation can be realized. Outside the region of optical trapping force, suspended microspheres follow Brownian motion in the solution until they are attracted and guided [56]. The videos of dynamic trapping of microspheres can be found in Supplementary Materials Videos S2–S4. As we can see from Figure 5e,f, with defocusing of the beam, the size of each lobe of the vertically accelerated 2D Airy beam increases, and the particles are separated according to the position of each lobe.

## 4. Discussion

Optical trap stiffness ($\kappa_{i}$) is evaluated to quantify the performance of optical trapping by the vertically accelerated Airy beam. According to the equipartition theorem and virtual spring method, the optical trap stiffness can be described as [58,59,60]

(3) $$\kappa_{i}=K_{B}T/\sigma_{i}^{2},$$

where *K_B_* denotes the Boltzmann constant, *T* is the absolute temperature in the ambience and $\sigma_{i}^{2}$ presents the position variance of a trapped particle along the *i* axis. As shown in Figure 5g–i, by tracking the spatial position of the captured microspheres, the *x-y* displacement from the equilibrium position of microspheres in the main lobe area is measured at different propagation depths. The corresponding position variance of trapped microspheres is calculated to determine the optical trap stiffness in the *x* and *y* directions at three different depths. In this work, we set K_B_ = 1.38 × 10^−23^ [(N∙m)/K] and T = 293 K. By the digital particle tracking method [61], the displacement of an attracted particle in the region of the main lobe can be recorded in the *x-* and *y-*direction at three different sample planes, respectively, and further calculated for the corresponding value of position variance of a trapped particle as presented in Table 1. According to Equation (3), the estimated optical trap stiffness in the *x-* and *y-*direction is presented in Table 2. In the intensity distribution of generated the vertically accelerated 2D Airy beam, the power inside the range of the main lobe accounts for ~13%, 11% and 3% of total incident input power (~170 mW) at *z* = 0.5 mm, *z* = 1.0 mm and *z* = 2.0 mm, respectively.

Owing to the ultrathin, compact, and flat design of metasurfaces, multiple optical trapping potentials can be realized for parallel guiding and sorting of particles [20,52]. Moreover, our metasurface based on beam shaper can be directly integrated with fiber optic probes and microfluidic devices [60,62]. Moreover, a tunable metasurface based on beam shaper can be designed to adjust trapping potentials along the axial direction [63,64,65]. Our technique is not limited only to Airy beams, and other exotic light beams can be created from a metasurface with different operation wavelengths, without any additional optical components.

## 5. Conclusions

In conclusion, a cubic-phase dielectric metasurface for the generation of polarization-independent vertically accelerated 2D Airy beam in the visible region is designed and experimentally demonstrated. The GaN-based metasurface with circular nanopillars provides many advantages, including high transmission efficiency, high refractive index to achieve 2π phase modulation and polarization-independent operation. The vertically accelerated Airy beam generated from our cubic-phase metasurface is characterized using FDTD simulation and experiments. The propagation trajectory of the generated vertically accelerated 2D Airy beam follows the theoretically predicted reciprocal trajectory. The unique optical characteristics of the vertically accelerated 2D Airy beam, such as non-diffraction, self-acceleration, and self-healing, are also experimentally verified. An experimental optical manipulation system equipped with the cubic-phase metasurface is configured to trap microspheres, both 3-μm and 1-μm microspheres, following the designed trajectory of the vertically accelerated 2D Airy beam at different axial planes. Due to the high-intensity gradients, microspheres are clustered along intensity patterns of the generated vertically accelerated 2D Airy beam and further guided for 3D manipulation. In addition, the performance of optical trapping has been evaluated by experimental measurement of trapping stiffness. The transverse and longitudinal manipulation range created by the cubic-phase metasurface is ~ 60 μm and ~ 2 mm, respectively, which can be adjusted by an input power or different NA objectives. The ultrathin size of the metasurface can provide novel possibilities for designing miniature optical manipulation systems for various biomedical applications.

## Figures and Tables

**Table 1:** The corresponding position variance of a trapped particle at different sample planes.

Position Variance	*z* = 0.5 mm	*z* = 1.0 mm	*z* = 2.0 mm
$\sigma_{x}^{2}$ [μm^2^]	0.0010	0.0014	0.0096
$\sigma_{y}^{2}$ [μm^2^]	0.0012	0.0016	0.0092

**Table 2:** The corresponding optical trap stiffness at different sample planes.

Position Variance	*z* = 0.5 mm	*z* = 1.0 mm	*z* = 2.0 mm
κ_x_ [pN/μm]	4.011	3.054	0.421
κ_y_ [pN/μm]	3.493	2.929	0.441

## Data Availability

The data presented in this study are available on request from the corresponding author.

## Supplementary Materials

The following are available online at https://www.mdpi.com/article/10.3390/nano11071730/s1, Table S1: The phase delay level and transmission of the unit cells with different diameters at 491 nm, Table S2: The phase delay level and transmission of the unit cells with different diameters at 532 nm, Table S3: The phase delay level and transmission of the unit cells with different diameters at 633 nm. Figure S1. The real and imaginary permittivity (ε) of GaN in the visible region, Figure S2: FDTD simulated intensity distribution profile of the designed cubic-phase metasurface along propagation direction at 532 nm under (**a**) x- and (**b**) y-polarized incident plane waves, respectively, Figure S3: (**a**) The experimental setup for measurement of the propagation properties of the vertically accelerated 2D Airy beam. The measured propagation trajectory of the generated beam in free space under (**b**) x- and (**c**) y-polarization illumination, Figure S4: The (**a**,**b**) simulated and (**c**,**d**) experimentally measured propagation trajectories of the generated vertically accelerated 2D Airy beam in free space at 491 nm and 633 nm. The white dash lines indicate the corresponding theoretical trajectory. Video S1: Dynamic movement of guided microspheres based on the generated vertically accelerated 2D Airy beam, Video S2: Dynamic trapping of microspheres at *z* = 0.5 mm, Video 3: Dynamic trapping of microspheres at *z* = 1.0 mm, Video S4: Dynamic trapping of microspheres at *z* = 2.0 mm.
