# Supplementary material for: Cubic-Phase Metasurface for Three-Dimensional Optical Manipulation
Source: Nanomaterials (Basel). 2021 Jun 30;11(7):1730. doi: 10.3390/nano11071730 (PMC8308168; doi:10.3390/nano11071730)
Supplement: Supplementary file 1 [file nanomaterials-11-01730-s001.zip › nanomaterials-1276694-supplementary.pdf]

Supplementary Materials

Cubic Phase Metasurface for Three-Dimensional Optical Manipulation

Hsin Yu Kuo [1,2,3,†], Sunil Vyas [3,†], Cheng Hung Chu [2,3], Mu Ku Chen [4], Xu Shi [5], Hiroaki Misawa [5], Yu-Jung Lu [1,2], Yuan Luo [3,6,7,*], Din Ping Tsai [1,2,4,*]

[1] Department of Physics, National Taiwan University, Taipei 10617, Taiwan; d06245003@ntu.edu.tw (H.Y.K); yujunglu@gate.sinica.edu.tw (Y.-J.L)
[2] Research Center for Applied Sciences, Academia Sinica, Taipei 11529, Taiwan; cheng-hung.chu@riken.jp
[3] Institute of Medical Device and Imaging, National Taiwan University, Taipei 10051, Taiwan; sunilvyas@ntu.edu.tw
[4] Department of Electronic and Information Engineering, The Hong Kong Polytechnic University, Hong Kong 999077, China; mu-ku.chen@polyu.edu.hk
[5] Research Institute for Electronic Science Hokkaido University, Sapporo 001-0021, Japan; shixu@es.hokudai.ac.jp (X.S); misawa@es.hokudai.ac.jp (H.S.)
[6] YongLin Institute of Health, National Taiwan University, Taipei 10672, Taiwan
[7] Department of Biomedical Engineering, National Taiwan University, Taipei 10051, Taiwan
* Correspondence: yuanluo@ntu.edu.tw (Y.L); dinping.tsai@polyu.edu.hk (D.P.T)
† These authors contributed equally to this work.

Note S1. Numerical Design of Unit Cells for Metasurface

To design unit cells for the metasurface, we used the commercial software Computer Simulation Technology (CST) Microwave Studio to compute the phase delay level and transmission for different diameters of GaN cylindrical nanopillars on the Al_2O_3 substrate. The pitch of the unit cell and the height of nanopillars are 250 nm and, 800 nm, respectively. The full 2π phase modulation is implemented by nine levels of phase gradient. The simulated phase delay level and transmission of each unit cell with different diameters at different operating wavelengths are shown in Table S1, S2 and S3, respectively, where the boundary conditions of x- and y-directions both are set in the unit cell model and the boundary condition of z-direction is employed with the opening model. The refractive index of the Al_2O_3 substrate is set as 1.77, and the complex relative permittivity of GaN is calculated using [1,2], which is as shown in Figure S1.

Table S1. The phase delay level and transmission of the unit cells with different diameters (D) at 491 nm.

Diameters (nm)	110	120	130	140	150	160	170	180	190
Phase delay level (rad)	0	0.618	1.428	2.336	3.236	4.259	5.293	5.627	6.656
Transmission (a.u.)	0.921	0.955	0.982	0.928	0.922	0.998	0.912	0.905	0.933

Table S2. The phase delay level and transmission of the unit cells with different diameters (D) at 532 nm.

Diameters (nm)	110	120	130	140	150	160	170	180	190
Phase delay level (rad)	0	0.473	1.006	1.628	2.409	3.277	4.029	4.738	5.530
Transmission (a.u.)	0.923	0.912	0.899	0.932	0.987	0.947	0.888	0.930	0.998

Table S3. The phase delay level and transmission of the unit cells with different diameters (D) at 633 nm.

Diameters (nm)	110	120	130	140	150	160	170	180	190
Phase delay level (rad)	0	0.276	0.596	0.981	1.460	2.015	2.579	3.103	3.623
Transmission (a.u.)	0.867	0.871	0.888	0.921	0.962	0.960	0.915	0.877	0.891

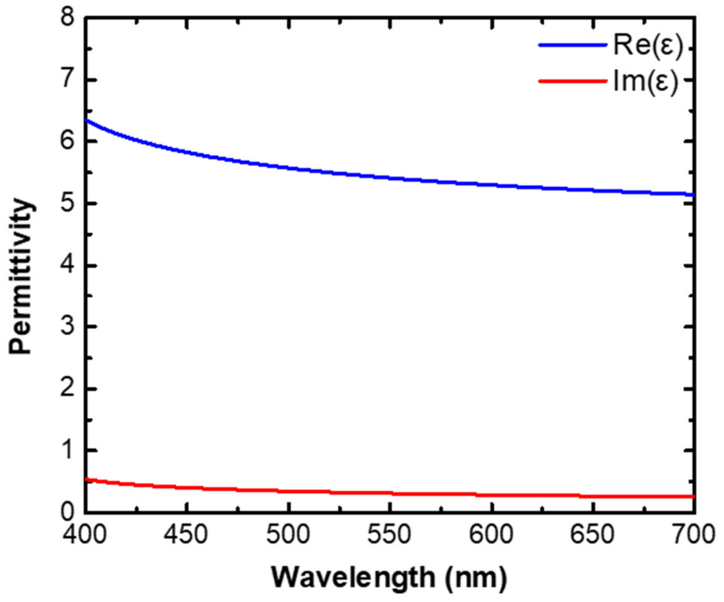

Figure S1. The real and imaginary permittivity (ε) of GaN in the visible region.

Note S2. Electromagnetic Simulated Methods in the Designed Cubic Phase Metasurface

The finite-difference time-domain (FDTD; Lumerical Solutions, Inc.) method is utilized to simulate the propagation characteristics of a vertically accelerated 2D Airy beam generated from the designed cubic phase metasurface, where the trajectory constant a is 0.033 mm^2. The volume of total simulated space is 30 μm x 30 μm x 150 μm (x, y, z) in which the boundary conditions are set for perfectly matched layers (PMLs) along three axes to minimize wave reflection. To verify the polarization-independent property of the designed metasurface, we set the input source with x- and y-polarized plane waves to generate a vertically accelerated 2D Airy beam

by the metasurface, respectively. As shown in Figure S2, whether illuminated by *x*- or *y*-polarized plane waves, the propagation trajectories of the generated vertically accelerated 2D Airy beams is identical and transmission efficiency in both cases are identical. The reason lies in the fact that the metasurface is composed of cylindrical nanopillars which are radially symmetrical and have the same electromagnetic responses under an arbitrary polarization source.

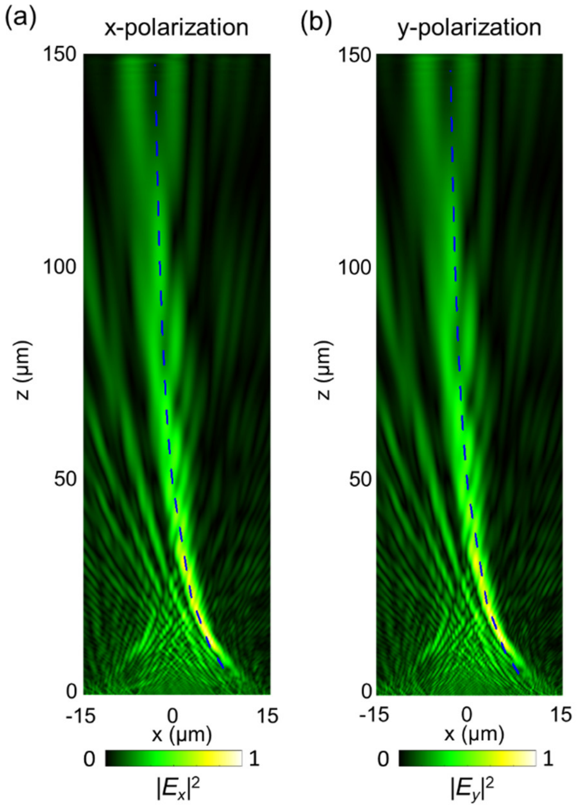

Figure S2. FDTD simulated intensity distribution profile of the designed cubic phase metasurface along propagation direction at 532 nm under (a) *x*- and (b) *y*-polarized incident plane waves, respectively. The blue dash line shows the theoretical propagation trajectory of the designed metasurface.

<u>Note S3. Experimental Results of the Polarization-Independence and Dispersion Properties of Fabricated Metasurface</u>

As shown is Figure S3a, the 532 nm laser source (Cobalt Samba™ 1500) is used to illuminate a cubic phase metasurface to generate a vertically accelerated 2D Airy beam in free space. Notably, we place the metasurface near the center of the incident Gaussian beam to cause the excitation source approximate to a plane wave. The objective lens (Mitutoyo, BD Plan Apo 5× magnification, NA = 0.14) and CCD (DSLR Camera Canon EOS 6D Mark II) both are placed on a linear motorized stage (NEWPORT GTS70) to measure the beam characteristics along the propagation direction. We put a linear polarizer in front of a metasurface to validate the polarization-independent property of the fabricated metasurface. As shown in Figures S3b and S3c, the measured propagation trajectories both present a similar reciprocal curve under *x*- or *y*-polarization illumination.

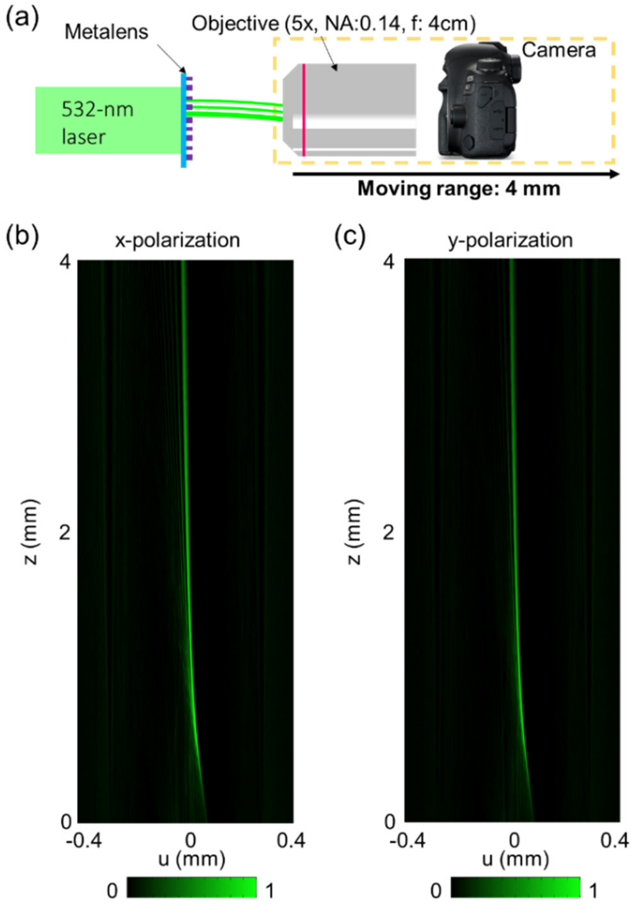

Figure S3. (a) The experimental setup for measurement of propagation properties of a vertically accelerated 2D Airy beam. The measured propagation trajectory of the generated beam in free space under (b) x- and (c) y-polarization illumination.

In this work, the phase distribution of the fabricated metasurface follows the original design at the operating wavelength of 532 nm and the simulated and measured propagation trajectory of generated 2D Airy beams at λ = 491 nm and 633 nm both deviate from the theoretical trajectory (white dash line), as shown in Figure S4. For the wavelengths other than the designed wavelength, the phase mismatching effect leads to less transmission efficiency and higher scattering of light noise.

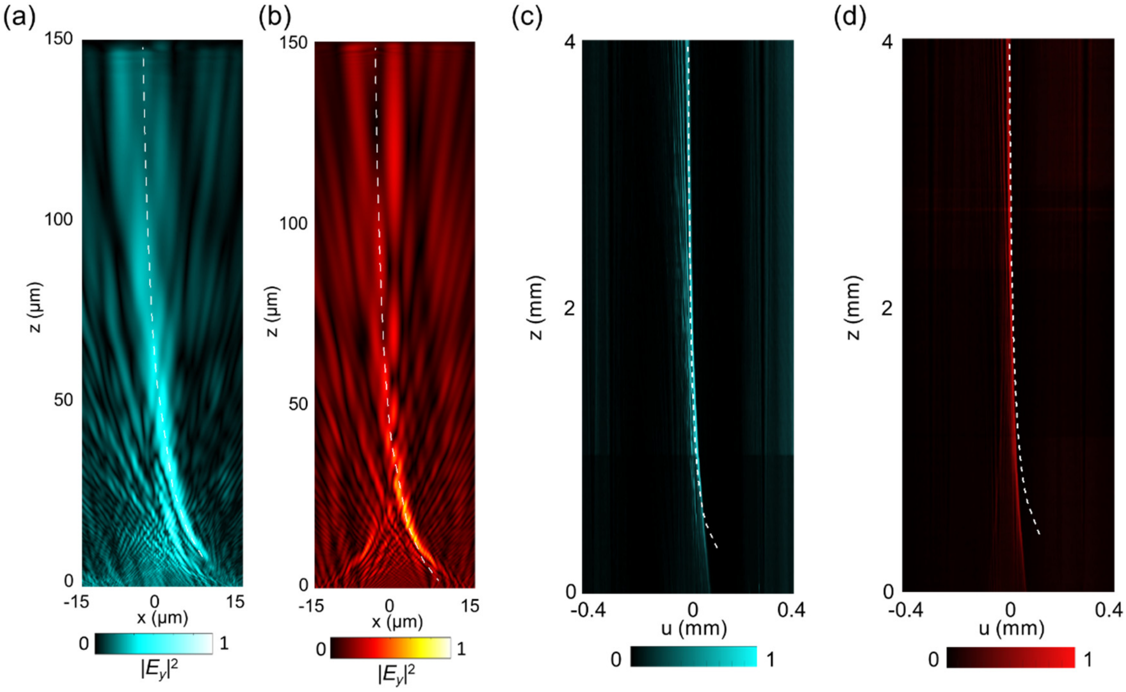

Figure S4. The (**a**,**b**) simulated and (**c**,**d**) experimentally measured propagation trajectories of the generated vertically accelerated 2D Airy beam in free space at 491 nm and 633 nm. The white dash lines indicate the corresponding theoretical trajectory.

References

1. Wang, S.M.; Wu, P.C.; Su, V.C.; Lai, Y.C.; Chen, M.K.; Kuo, H.Y.; Chen, B.H.; Chen, Y.H.; Huang, T.T.; Wang, J.H; et al. A broadband achromatic metalens in the visible. *Nat. Nanotechnol.* **2018**, *13*, 227–232.
2. Kawashima, T.; Yoshikawa, H.; Adachi, S.; Fuke, S.; Ohtsuka, K. Optical properties of hexagonal gan. *J. Appl. Phys.* **1997**, *82*, 3528–3535.
